# Women in Medicine, Law and Theology

**Published:** 1875-11

**Authors:** 


*Women in Medicine, Law and Theology.*

Amongst the burning questions of the day is the propriety or the right of admitting women into the professions. I say the right or propriety, because even if the right were made good, the propriety would not follow. Why it is that the women have selected medicine as the especial point of attack is not quite clear. They are far better fitted, by organization, by natural aptitude, to shine in the pulpit and at the bar; and in the career of arms, if we may trust legendary or mystic history, they have earned no mean distinction. But medicine, whilst demanding physical power not less than the other professions, is essentially based upon science.

Now, the women who have been distinguished for scientific power might be counted on the fingers of one hand. There seems to be a natural incompatibility between science and the female brain. There is, indeed, no such inherent incompatibility between science and religion or law. Theology and law that are not in harmony with true science must rest upon a very insecure foundation. But the clergy of all denominations, and lawyers as a rule, assume a direct antagonism to science. They set themselves above it; they would trample it down as something in chronic rebellion against their authority. In this antagonism they resemble the women; in these they find their most useful allies. The church and the law, then, are the professions most congenial to the somewhat arbitrary character of the female intellect.—Robert Barnes *in Obstetrical Journal,* August, 1875.

The great Author of our being never intended that one sex should usurp the prerogatives—the native rights—the duties of the other. Harmony is the law of nature; and the unison, the concord of creation, is preserved by an admirable distribution of qualities and powers. And so it is in relation to man and woman, in which, more strikingly than in any other of God's creation, the wisdom of His divinity is apparent.

Man is strong—woman is beautiful; man is daring and confident—woman is diffident and unassuming; man is great in action—woman in suffering; man shines abroad—woman at home; man talks to convince—woman to persuade and please; man prevents misery—woman relieves it; man has science—woman, taste; man has judgment—woman, sensibility; man is a being of justice—woman, of mercy.

Far be it from me to attempt to depreciate her virtues and her worth. Her power and influence for the happiness of man, for the good of society, for the prosperity of the State, and for the progress of religion, are (although exerted in a different sphere) fully equal to that of the opposite sex.

It is a truism that, in whatever capacity we view the true woman, we find her vindicating the truth of Holy Writ by proving herself a help-meet to man. Great is her power for good, and great is our appreciation of her worth. To woman the civilized world pays the tribute of its admiration; aye, and the measure of that admiration is, by common consent, regarded as the measure of refinement and moral elevation. Woman needs not now, as in the dark ages of the past, the aid of gallant knight, with lance in rest, to proclaim her merits, or to declare her mistress of our hearts, or to protect her from insult or danger. Nay, to her our affections point as the pole-star of our life, the partner of our joys and of our sorrows, as we traverse this "vale of tears," and as our mentor to point us unerringly to the path of duty, and to the great recompense of reward that lies beyond.

Our rhetoricians, when personifying the sun, have placed it in the masculine gender, and have by rhetorical rule awarded the feminine gender to the moon; because (as they say) the latter orb displays only a borrowed light, and is tributary to the great center of all the heavenly bodies. In this instance, I beg leave to reverse the metaphor, for the reason that the dutiful daughter, the true wife, the mother of a household shines forth with a purely original light, and, in her domestic sphere, she is really a source from whence emanate all those rays of virtue and happiness which characterize the Christian family. As the daughter, she is one of the shining stars in the galaxy of the domestic virtues, the pride of the father and brother, the solace and comfort of the mother. It is in a daughter's heart that filial love takes sweetest and deepest root, and in its development acquires all the force and potency of a fixed law and principle of action. The ardor of a daughter's affections is ever constant. When sickness blanches the cheek and dims the eye of a beloved parent, the daughter watches by the bedside, and never leaves it until the bloom of health returns, or the features are rigid in death. The daughter is the fragrant and immortal flower in which the sacred tenderness of love finds its most beautiful personification, and whose sweetness is the incense that rises unceasingly from the altar of home to the God of love and nature.

As the wife, she is the guardian angel of man—the bright, particular star of his earthly elysium. She is the potent source of that bliss which has alone survived, unblemished, the sinful fall of our first parents' domestic happiness. Her might is gentleness; the scepter of her sway is a soft word or a softer look. What her gentleness and love cannot encompass must remain forever unattainable.

As majesty, power and strength are the glorious attributes of man, so is sweetness the imperishable attribute of woman, by which she reigns supreme, especially in the family circle. She reigns queen over the empire of the beautiful, and is acknowledged sovereign of loveliness. And the possessor of such a treasure, an honest, true-hearted, loving wife, may indeed exclaim with the ancient philosopher: "Eureka! Eureka!" for the treasures of the deep are not so precious as are the concealed comforts of a man locked up in woman's love.

As a mother, she has the power to preserve the powerful image of Heaven in the soul of the child, and become the instructor of mankind. The lessons she teaches are never forgotten. Blended with her sweet image, they recur to memory in remotest years, and the lip of a[illegible]murs the prayer learned upon her knee. She instills truth, justice, integrity, and every other noble and honorable sentiment. To have had a good mother is the stepping-stone to honorable success in this life, and an immortality of bliss in the other. Faithful herself to the sacred trust imposed upon her by Heaven, she looks upon the future life of her offspring with just pride and pleasure, and can, with fond satisfaction, exclaim, in the words of the noble Roman matron: "These are my jewels!" She has the consciousness that she has not lived in vain. Her remembrance is cherished as a holy thing, when she has passed the dark river of death to her final reward beyond, in the fair fields of Eden. The honor of her sons, the love and piety of her daughters, is the pledge that she shall at length present herself and them, faultless and unblemished, before the throne of God with exceeding joy.

Who but woman is adequate to these high responsibilities—to the task of presiding with true dignity over the interests which connect themselves so intimately with time and with eternity? Ah! she is, indeed, like a "vine planted by Jehovah, which shall send out its branches, bearing fruit to many generations"—like unto stars illuming the pathway of life, and whose sweet influences keep our feet therein. This is at last the glorious field of her noblest achievements, and where her rarest virtues have the purest, brightest, and most enduring luster. This is her true sphere of life, where all her best and noblest attributes are exercised for the well-being and happiness of the human race.

Oh! what a priceless blessing is such a woman! But, alas! whilst history commemorates its thousands, it holds up a host who have been recreant to their high and holy duties, and have sacrificed all which dignifies woman—all which constitutes her true enjoyment.

Oh! woman! the "path of duty is the path of safety," in the moral as in the natural world. According to what you sow, that shall you reap. Therefore, to my medical brethren I would say, forget not the eternal obligations you owe to woman; and if you can do no more, advise your female friends to study the dark as well as the bright phases of woman's career, as recorded in the pages of history, before they enter either the field of medicine, law or theology.

